# Diagnostic and Prognostic Utility of a DNA Hypermethylated Gene Signature in Prostate Cancer

**DOI:** 10.1371/journal.pone.0091666

**Published:** 2014-03-13

**Authors:** Liang Kee Goh, Natalia Liem, Aadhitthya Vijayaraghavan, Gengbo Chen, Pei Li Lim, Kae-Jack Tay, Michelle Chang, John Soon Wah Low, Adita Joshi, Hong Hong Huang, Emarene Kalaw, Puay Hoon Tan, Wen-Son Hsieh, Wei Peng Yong, Joshi Alumkal, Hong Gee Sim

**Affiliations:** 1 Centre for Quantitative Medicine, Duke-National University of Singapore Graduate Medical School, Singapore, Singapore, Singapore; 2 Cancer & Stem Cell Biology, Duke-National University of Singapore Graduate Medical School, Singapore, Singapore, Singapore; 3 Cancer Science Institute, National University of Singapore, Singapore, Singapore, Singapore; 4 Department of Urology, Singapore General Hospital, Singapore, Singapore, Singapore; 5 Department of Pathology, Singapore General Hospital, Singapore, Singapore, Singapore; 6 Knight Cancer Institute, Oregon Health and Science University, Portland, Oregon, United States of America; Deutsches Krebsforschungszentrum, Germany

## Abstract

We aimed to identify a **p**rostate cancer DNA **hy**permethylation **m**icro**a**rray signature (denoted as **PHYMA**) that differentiates prostate cancer from benign prostate hyperplasia (BPH), high from low-grade and lethal from non-lethal cancers. This is a non-randomized retrospective study in 111 local Asian men (87 prostate cancers and 24 BPH) treated from 1995 to 2009 in our institution. Archival prostate epithelia were laser-capture microdissected and genomic DNA extracted and bisulfite-converted. Samples were profiled using Illumina GoldenGate Methylation microarray, with raw data processed by GenomeStudio. A classification model was generated using support vector machine, consisting of a 55-probe DNA methylation signature of 46 genes. The model was independently validated on an internal testing dataset which yielded cancer detection sensitivity and specificity of 95.3% and 100% respectively, with overall accuracy of 96.4%. Second validation on another independent western cohort yielded 89.8% sensitivity and 66.7% specificity, with overall accuracy of 88.7%. A PHYMA score was developed for each sample based on the state of methylation in the PHYMA signature. Increasing PHYMA score was significantly associated with higher Gleason score and Gleason primary grade. Men with higher PHYMA scores have poorer survival on univariate (p = 0.0038, HR = 3.89) and multivariate analyses when controlled for (i) clinical stage (p = 0.055, HR = 2.57), and (ii) clinical stage and Gleason score (p = 0.043, HR = 2.61). We further performed bisulfite genomic sequencing on 2 relatively unknown genes to demonstrate robustness of the assay results. PHYMA is thus a signature with high sensitivity and specificity for discriminating tumors from BPH, and has a potential role in early detection and in predicting survival.

## Introduction

Prostate cancer is the most incident cancer in the United States with an estimated new 238,590 diagnoses (153 cases per 100,000 per year). As a cause of death, it is estimated at only 29,720 cases (12%) [1]. This discordance is seen also in Singapore, Japan and Korea, where the ratio of incidence to mortality is approximately 0.2 [2] and has largely been attributed to over-diagnosis of clinically insignificant prostate cancers as a result of widespread prostate-specific antigen (PSA) testing.

The increase in detection of early prostate cancer has not been accompanied by accurate determination of risk for morbidity and mortality. This has resulted in over-treatment in some men and under-treatment in others. Gleason grading, a low-power microscopic evaluation of prostate cancer architecture described in 1966 by Donald Gleason, has remained the mainstay of prostate cancer prognostication [3], [4]. However, the grading system, is subject to inter-observer differences and lacks precision in prognosticating early stage prostate cancers which are increasingly being diagnosed [5], [6]. This has been partially addressed by modifications [7], inclusion of tertiary scores [8] and the use of clinical nomograms [9]. Marked stage migration to 80% of prostate cancers being diagnosed at an organ-confined stage has blunted the efficacy of these tools at the point of diagnosis. Active surveillance, a strategy of selectively delaying radical treatment in very low risk early prostate cancer, involves frequent follow-up and annual prostate biopsies - a source of considerable anxiety and cost [10]. While other blood and tissue biomarkers to prognosticate prostate cancer are in development, few of them have been extensively validated and none of them are in clinical use [11].

Epigenetic changes, which involve mechanisms that initiate and maintain heritable patterns of gene expression without altering the sequence of the genome, is a process with several layers of complexity [12]. These include histone modifications, chromatin remodeling, nucleosome occupancy and DNA methylation. Of these mechanisms, DNA methylation is the most well-described and studied. Promoter hypermethylation and consequent transcriptional silencing has been found to be widespread and associated with hundreds of genes in almost all cancer types and has emerged as an important focus of epigenetic research [12], [13], [14]. Evidence from earlier studies has linked promoter hypermethylation of specific genes to pathogenesis and tumor progression in prostate cancer, e.g. *APC, RARβ*, and *GSTP1* [15]. However, these studies focused on selected methylated genes rather than global gene methylation, which limit the sensitivity and specificity for diagnosis and prognosis. A diagnostic or prognostic signature involving multiple genes can improve discriminatory power. Although molecular signature from gene expression arrays showed promising sensitivity (93–97%) and specificity (87–100%) [16], [17], the stability of DNA material makes hypermethylation signature a more viable option, especially for archived paraffin-embedded tissue samples. While a number of studies have utilized genome wide methylation arrays [18], [19], [20], [21], [22], the findings are still limited to candidate genes approach. Rapid progress in epigenetics has enabled the development of high throughput techniques to analyze the methylation patterns of prostate cancer genes to elucidate molecular signatures that can be surrogate for the Gleason grading system.

Our goal was to identify unique global gene hypermethylation changes that were cancer-specific and that would discriminate different prostate cancer phenotypes. We used high-throughput DNA methylation microarrays and analyzed a large number of gene loci in human prostate cancer tissues with the aims of identifying diagnostic DNA hypermethylation signature that differentiates prostate hyperplasia (BPH), low grade and high grade prostate cancers with a view to distinguish lethal from non-lethal prostate cancers.

## Materials and Methods

### Study Design and Patient Inclusion

Approval for this study was provided by SingHealth Centralized institutional Review Board B (Approval number CIRB #32B/2007). The need for consent was waived by the review board in view of the retrospective nature of the study and long archival period of the tissues involved.

This is a non-randomized retrospective study of gene hypermethylation in prostate cancer and benign prostate hyperplasia (BPH). Cases of prostate cancer were stratified based on stage and grade. Clinical and pathological staging was performed using the 2007 AJCC TNM staging and histological grading was based on the Gleason grading system [7].

Paraffin embedded archival tissues of local Asian population was obtained from single institution (Singapore General Hospital). Tissues for cancer cases were obtained from patients with early clinically localized prostate cancer and advanced metastatic prostate cancers. Tissues from the former group were obtained from men who underwent radical prostatectomy; tissues from the latter group were obtained from men who underwent transurethral resection of prostate and bilateral orchiectomy at the same time. Regional lymph node metastasis was detected by abdomen-pelvic CT and MRI scans and bony metastatic disease was confirmed by positive Technetium^99^ bone scans. Patients who underwent radiation or androgen deprivation therapy before transurethral resection were not included. BPH cases were obtained from transurethral resection in men with benign prostate enlargement and PSA <4 ng/ml pre-operatively and benign status was confirmed histologically. Control tissues were obtained from BPH, as BPH is common in the cohort of men susceptible to prostate cancer and it remains the most common differential diagnosis in these men. We did not use adjacent normal tissue in men with prostate cancer to avoid any possible field change effects [23].

### Specimen Collection

The grade and stage of each specimen was first confirmed under haematoxylin and eosin stain by the pathologist. Cancer rich areas were marked and the prostate epithelia isolated by laser capture microdissection techniques using the Zeiss P.A.L.M. system (Carl Zeiss MicroImaging GmbH, Jena, Germany). Patient demographic and tumor profile, including age, pre-operative PSA level, clinical and pathological stage, and pathological Gleason grade were obtained. Duration of follow-up, and time to biochemical recurrence and cancer specific mortality were recorded.

### DNA Extraction and Sample Profiling

Genomic DNA was extracted using 300 µL of digestion buffer solution (50 mM Tris-HCl, 1 mM EDTA, 0.5% Tween 20) with 20 µL of Proteinase K (20 mg/ml, Qiagen, Valencia, CA). The samples were incubated at 55°C for 48 hours in a rotating oven. Proteinase K was inactivated after incubation at 94°C for 10 minutes and centrifuged briefly for 5 minutes at 260 *g*. The quantity of extracted DNA was assessed by real-time PCR as previously described [24]. To optimize DNA conversion and reduce variability between samples, 500 nanograms of genomic DNA was bisulfite-converted using EZ DNA Methylation kit (Zymo Research, Orange, CA) as per manufacturer’s recommendations. Bisulfite conversion of genomic DNA results in unmethylated cytosine being converted to uracil while methylated cytosines remain unchanged. The samples were then profiled on the Illumina GoldenGate BeadArray according to manufacturer’s specifications. Briefly, bisulfite-converted DNA undergoes hybridization to allele specific oligonucleotide and locus-specific oligonucleotide that is dependent on the C/T nucleotide polymorphism at each CpG site. Fluorescence labeled PCR primers of Cy3 was used to amplify unmethylated template DNA, whereas Cy5 labeled primers was used to amplify methylated template DNA. The intensity of the 2 fluorescence signals was analyzed on a Sentrix Array Matrix, and the level of methylation at the CpG locus was measured by the β value where β = [max (Cy*5*, 0)]/(|Cy*3*|+|Cy*5*| +100). The β value was an average of multiple measurements of 30 replicates taken at each CpG loci and results range between 0–1.0 after subtracting the background provided by internal controls within the array matrix [25]. For quality control, universal methylated and unmethylated samples were included as controls in the profiling. To ensure data quality, samples with high bisulfite conversion ≥20,000 were included in the study.

### Bisulfite Sequencing to Validate Novel Genes

DNA extraction and bisulfite modification of DNA was described above. Bisulfite genomic sequencing (BGS) analyses for *ALOX12* and *PDGFRB* were performed with the following primer sets: For *ALOX12*: BGS5: GGGAGGTTTAGGAAGGTTT; BGS6: AAAACTAACTATACCTCCTAATC. For *PDGFRB*: BGS2: AATCTCCCTAAATACCATAACAA; BGS3: GGGATGTTTAGAAATTTTATAGTT. All primer sets were previously tested for not amplifying any unbisulfited DNA. For BGS, all PCR reactions were carried out using the AmpliTaq-Gold DNA polymerase for 40 cycles with “hot start” (Applied Biosystems, CA, USA). The PCR products were gel-purified, excised and TA-cloned into the pCR2.1-TOPO vector (Invitrogen, CA, USA) and 8–12 colonies were randomly chosen and analyzed using dideoxynucleotide sequencing.

### Generation of Classification Model

Raw files from the Illumina platform were processed using Illumina GenomeStudio to obtain β values (range from 0 to 1) for each probe and sample. The β is an indication of observed methylation relative to the maximum potential methylation of each probe. 111 samples (87 cancers, 24 BPH) were successfully profiled, passed the quality control (see sample profiling), and were available for analysis. To generate a classification model that will differentiate prostate cancer and BPH without over fitting the data, a 2-stage design of training (discovery) and testing (validation) was employed [26], [27]. The 111 samples were randomly stratified into training (Tumor = 44, BPH = 12) and testing (Tumor = 43, BPH = 12) datasets, while maintaining original prevalence of disease stage as specified by Gleason score in both datasets. This was to ensure both datasets have comparable distribution on disease stage. Analysis was confined to hypermethylated probes, i.e. probes where mean β value of tumors was greater than mean β value of BPH [(µ_tumor_−µ_control_)>0] in the training dataset. This resulted in 1013 probes for modeling. Support vector machine (SVM, http://www.chibi.ubc.ca/gist/index.html) [28], [29] was used to generate the classification model on the training dataset using the following parameters: (i) recursive feature elimination for probes selection, (ii) radial basis kernel function with σ = 1, (iii) reduction of probes per iteration = 10%, and (iv) number of iterations = 40. The resultant SVM model was then validated on the testing dataset, where performance was evaluated on sensitivity, specificity, overall accuracy, and receiver operating characteristics (ROC), which assesses area under the curve for performance.

### Independent Dataset

Independent validation was performed on a western cohort obtained from Oregon Health & Science University (OHSU) [18]. Baseline characteristics of the cohort are summarized in Table S1 in File S1. The raw data was processed similarly as described in Methods. 59 tumors and 3 normal (patients without histological evidence of prostate cancer) prostate samples passed the quality control (see sample profiling) and were used for validation. Clinical data including Gleason score, stage, and recurrence were available and evaluated.

### PHYMA Signature and Score

The hypermethylated probes elucidated from the SVM modeling was denoted as the **PHYMA** signature – i.e. **p**rostate cancer DNA **hy**permethylation **m**icro**a**rray signature. To assess association of PHYMA signature with clinicopathological factors, we translated the PHYMA signature into a single index which we denote as a PHYMA score by counting the number of probes in PHYMA signature with β≥0.7. This was to facilitate clinical interpretation of the PHYMA signature via a single prognostic factor.

The threshold of β≥0.7 was determined by inspection of the universal methylated and unmethylated control assays (see quality control protocol in sample profiling). It was observed that β≥0.7 was significantly robust to differentiate between methylated and unmethylated (p<2.2e-16, Fisher’s exact test), and was thus used to compute the number of probes (out of the 55) that were methylated for each sample. In another study on DNA methylation of bone marrow cells [30], it was found median β for hypermethylation was 0.75, thus further supporting the threshold used in this study.

### Data Analyses

Clinical relevance of PHYMA score with Gleason indices (i.e. Gleason score and Gleason primary grades) was assessed using linear regression. Analyses for PHYMA scores between tumor and BPH, low (Gleason score 6) and high (Gleason score 8–10) grades were performed with Welch’s t-test. Overall survival or biochemical recurrence-free survival was analyzed with Kaplan-Meier and Cox proportional hazard (Wald test) for univariate and multivariate models respectively, with clinical stage and Gleason scores as covariates in the multivariate models. In survival analyses, samples were stratified into low and high PHYMA scores based on median of PHYMA scores of our dataset (i.e. High PHYMA group: PHYMA score>32, Low PHYMA group: PHYMA score< = 32). Overall survival was calculated from the date of diagnosis to the date of death due to the disease. Men who were alive or lost to follow-up at the time of analysis were censored at the date of last follow-up. Statistical analyses were computed using R statistical and survival packages (www.r-project.org). Pathway analysis was performed using Ingenuity Pathway Analysis software (Ingenuity, California, USA). The methylation dataset is MIAME compliant and has been submitted to Gene Expression Omnibus (GEO) website, series accession number GSE39603. The link to the dataset is http://www.ncbi.nlm.nih.gov/geo/query/acc.cgi?token=zzylboguukqgqpm&acc=GSE39603.

## Results

A total of 111 paraffin-embedded archival tissue samples obtained from 87 men with prostate cancer and 24 men with BPH from surgery performed at a single institution between 1995 and 2009, and 1013 hypermethylated probes from the Illumina array were used for this study. Almost three quarters of the cancer cases have clinically organ-confined cancer (T1 42.5%, T2 25.3%) and about half had high Gleason grade disease (Gleason score ≥8 55.2%). A quarter of cases were metastatic at the time of presentation (M1 25.3%, M0 74.7%), of which one case had underwent radiation and hormone therapy prior to tissue acquisition during channel TURP (transurethral resection of prostate). Table 1 summarizes the baseline characteristics of these cancer patients.

**Table 1:** Baseline characteristics of 87 prostate cancer and 24 BPH patients included in the study.

	Prostate Cancer (n = 87)	BPH (n = 24)
Age at Diagnosis, years		
Median (range)	68 (52–94)	71(50–81)
Follow up, months		
Median (range)	48(0–175)	53.5(1–59)
PSA, ng/mL, median (range)	28.7±326.7	7.1(0.73–38.8)
Gleason Score		
G6, n (%)	19 (29.2)	–
G7, n (%)	20 (23.0)	–
G8–G10, n (%)	48 (55.2)	–
Clinical T Stage (n = 87)		
TX, n (%)	5 (5.8)	NA
T1, n (%)	37 (42.5)	NA
T2, n (%)	22 (25.3)	NA
T3, n (%)	16 (18.4)	NA
T4, n (%)	7 (8.0)	NA
M1 disease	22 (25.3)	NA

### Classification Model for Differentiating Tumors and BPH

**Figure 1:**
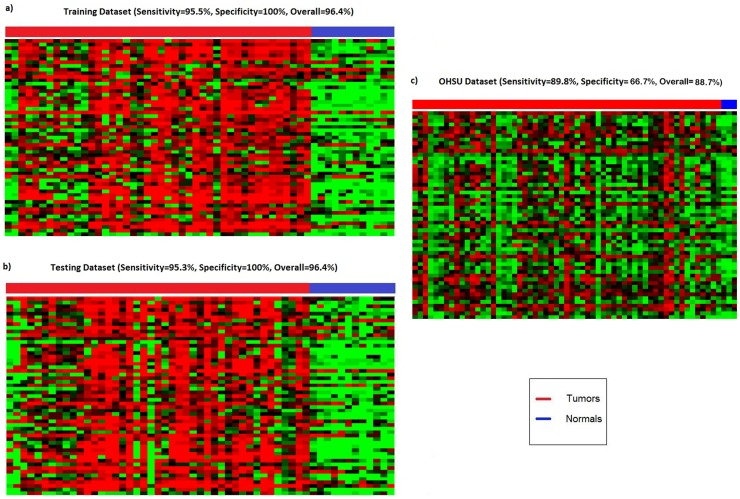
Heatmaps of PHYMA signature in Asian and western datasets. (a) Training dataset of local Asian population. (b) Testing dataset of local Asian population. (c) OHSU western cohort dataset. Each row represents a methylation probe and column a sample. The level of methylation varies from green (low β) to red (high β). Tumor samples showedmore aberrant DNA methylation compared to BPH tissue. All tumor samples showed comparatively higher DNA methylation compared to the non-tumor samples.

Classification model was generated using a 2-stage study design of training and testing. Using random sampling, both training and testing datasets have comparable distribution in terms of sample sizes, stage and grade for tumors and BPH. A SVM model was generated using a signature consisting of 55 probes targeting CpG loci of 46 genes; a hypermethylation signature which we denote as “**PHYMA**”. Figure 1 shows corresponding heatmaps for training and validation datasets. In the training dataset, there was a high level of sensitivity and specificity, 95.5% (42/44) and 100% respectively (Figure 1a). The first validation dataset (i.e. testing dataset) yielded similar performance; with 95.3% (41/43) sensitivity and 100% specificity (Figure 1b). A second validation on an independent OHSU dataset showed 53/59 (89.8%) tumor and 2/3 normal (66.67%) samples were correctly classified (Figure 1c). In the training dataset, 2 tumors were misclassified giving overall accuracy of 96.4%. For validation datasets, 2 tumors were misclassified for testing dataset and 6 tumors in the OHSU dataset. All BPH samples were classified correctly in training and testing datasets, while 1 normal sample was misclassified in OHSU. The 2 validation datasets of testing and OHSU gave overall accuracy of 96.4% (ROC = 0.998) and 88.7% (ROC = 0.927) respectively. This finding support robustness of the PHYMA signature and classification model. The capability of the signature to differentiate tumors from BPH samples in validation datasets demonstrates the underlying biological relevance of the genes in PHYMA in prostate cancer. Table S2 in File S1 lists the 55 probes and its corresponding weight in the SVM model.

### Clinical Relevance of PHYMA

It was evident in Figure 1 that cancer samples have more aberrant DNA methylation compared to BPH. The PHYMA signature of BPH samples showed comparatively reduced methylation across the probes. This is in concordance with literature that cancers tend to harbor more methylation when compared to non-cancerous tissue [31].

**Figure 2:**
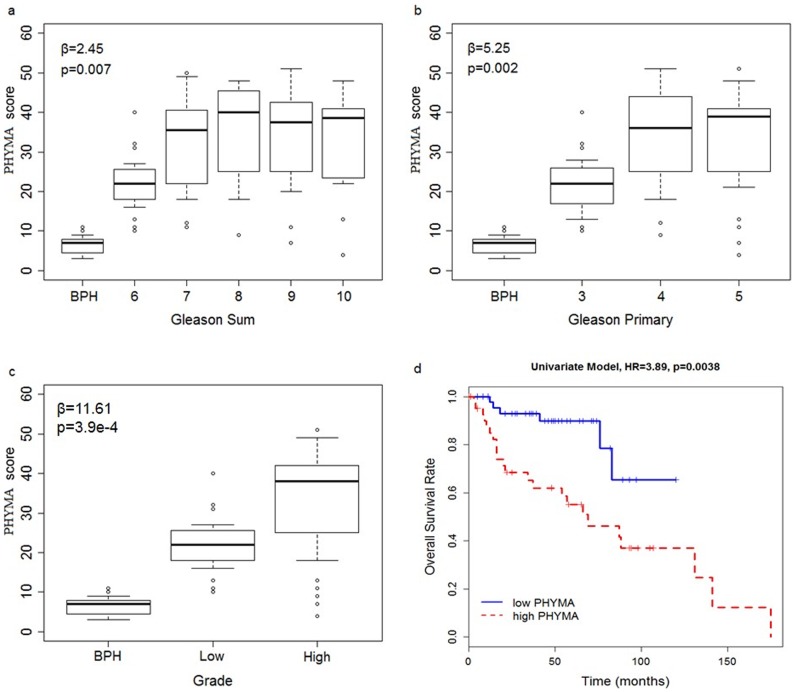
Association of PHYMA scores with clinical factors. (a) Gleason score, (b) Gleason primary grade, (c) difference between low and high grade, and (d) overall survival. Tumor samples have higher PHYMA scores compared to BPH and are associated positively with (a) Gleason score, (b) Gleason primary grade, and (c) difference between high and low grades. (d) Kaplan Meier show samples with higher PHYMA have poorer survival.

Using PHYMA score of each sample, clinical relevance with respect to Gleason scores and overall survival was evaluated. Figure 2 shows association of PHYMA with each of the clinical parameter for the combined training and testing dataset. As expected, cancer samples had higher PHYMA score than BPH (p<2.2e-16, Figure 2a). Generally, cancers with Gleason primary grade 3 have lower PHYMA scores, while higher Gleason primary grades showed higher PHYMA scores, suggesting that aberrant methylation of these 55 probes could be surrogate for histological grade. Regression analysis on tumor samples indicated PHYMA scores were associated positively with Gleason score (beta = 2.45, p = 0.007, Figure 2a), Gleason primary grade (beta = 5.25, p = 0.002. Figure 2b), and difference between low and high grades (beta = 11.61, p = 3.9e-4, Figure 2c). Analysis on PHYMA scores showed high Gleason grade tumors have higher PHYMA score than low grade tumors (p = 1.59e-5, Figure 2c). Using median of PHYMA scores of 32 as a cut-off, tumor cases were divided into high and low PHYMA scores groups. Overall survival univariate analysis revealed patients in the high PHYMA scores group has poorer survival (p = 0.0038, Wald test, HR = 3.89, Figure 2d & Table 2 Model 1). Multivariate overall survival analysis controlling for (i) clinical stage (Model 2: p = 0.055, HR = 2.57), (ii) clinical stage and Gleason scores (Model 3: p = 0.043, HR = 2.61), showed high PHYMA scores group was associated with poorer survival outcome regardless of Gleason scores (Table 2). Clinical stage and Gleason scores are known prognostic indicators for prostate cancer. Our study thus showed that PHYMA score can be a potential independent indicator and can complement prognosis prediction of the disease. Taken together, these results showed significant association of PHYMA scores with histological grade, and PHYMA scores may be able to differentiate between lethal and non-lethal tumors regardless of Gleason indices.

**Table 2:** Cox proportional hazard models for overall survival.

	Regression Coefficient	Standard Error	Hazard Ratio	95% CI for hazard ratio	p-value	Concordance Index
**Model 1 (univariate)**						
PHYMA	1.36	0.47	3.89	1.55–9.72	0.0038*	0.677
**Model 2 (multivariate)**						
PHYMA	0.94	0.49	2.57	0.98–6.72	0.055	0.763
Stage	0.68	0.21	1.96	1.31–2.95	0.0012*	
**Model 3 (multivariate)**						
PHYMA	0.96	0.47	2.61	1.03–6.61	0.043*	0.822
Stage	0.43	0.22	1.54	1.00–2.39	0.052	
Gleason score	0.63	0.20	1.87	1.28–2.74	0.0013*	

Univariate and multivariate models were evaluated with clinical stage and Gleason score as covariates.

*statistical significance, p<0.05.

Evaluation of clinical relevance on OHSU samples showed tumors in this western cohort tend to have lower PHYMA scores compared to our Asian cohort (Figure S1a in File S1), likely attributable to cohort effects such as experimental and/or ethnic differences. Similar to our Asian cohort, OHSU tumors harbored higher PHYMA score than normal samples. Regression analyses on PHYMA scores did not show significance though similar positive trend was observed from Gleason score 4 to 8 (beta = 2.28, p = 0.2). Interestingly, PHYMA scores for Gleason score 9 showed a dip compared to Gleason score 8, as in our data. Of 6 incorrectly classified tumors, most were of lower Gleason score; 4 from Gleason score 6, 1 from Gleason score 7, and 1 from Gleason score 8. Only recurrence information was available for OHSU dataset and recurrence analyses revealed similar trend as our overall survival although no significance was observed (p = 0.369); higher PHYMA scores tumors tend to have poorer prognosis (Figure S1b in File S1). It should be noted that the sample size for the recurrence analysis is small, n = 18.

### Biological Relevance of PHYMA

Several studies have utilized high-throughput microarray technology to identify hypermethylated genes in prostate tumors [18], [19], [20], [21], [22]. Despite different array platforms, majority of the genes in PHYMA signature (42/46, 91.3%) were reported in these studies (Table S3 in File S1). Some of the genes like *GSTP1, GSTM2, APC, RARB, TJP2, SEPT9,* and *ADAMTS12* were found to be reported in more than one of the above mentioned studies. This shows robustness of our results and further supports functional role of the genes in PHYMA signature.

Pathway analyses showed the 46 genes in PHYMA were biologically relevant in cancer and reproductive system disease, specifically in functional roles such as cellular movement, cell-to-cell-signaling and interaction, and inflammatory response. Majority of the genes were previously implicated in prostate cancer, such as: *ALOX12, APC, CDKN1B, EPHA2, GSTP1, KIT, MMP7 MMP9, PDGFRB, PYCARD, RARA, RARB, RARRES1*, and *TIMP1* [32], [33], [34]. Several are known biomarkers for diagnosis and prognosis of prostate cancer (*APC, GSTP1, KIT, PYCARD, PGDGRB, RARB, RARRES1*, and *TIMP1*) [35], [36], [37], [38], [39], [40] and some though not biomarkers, were found to be dysregulated in the disease (*CDKN1B, MMP7*, and *MMP9*) [41].

### Methylation Status of ALOX12 and PDGFRB

**Figure 3:**
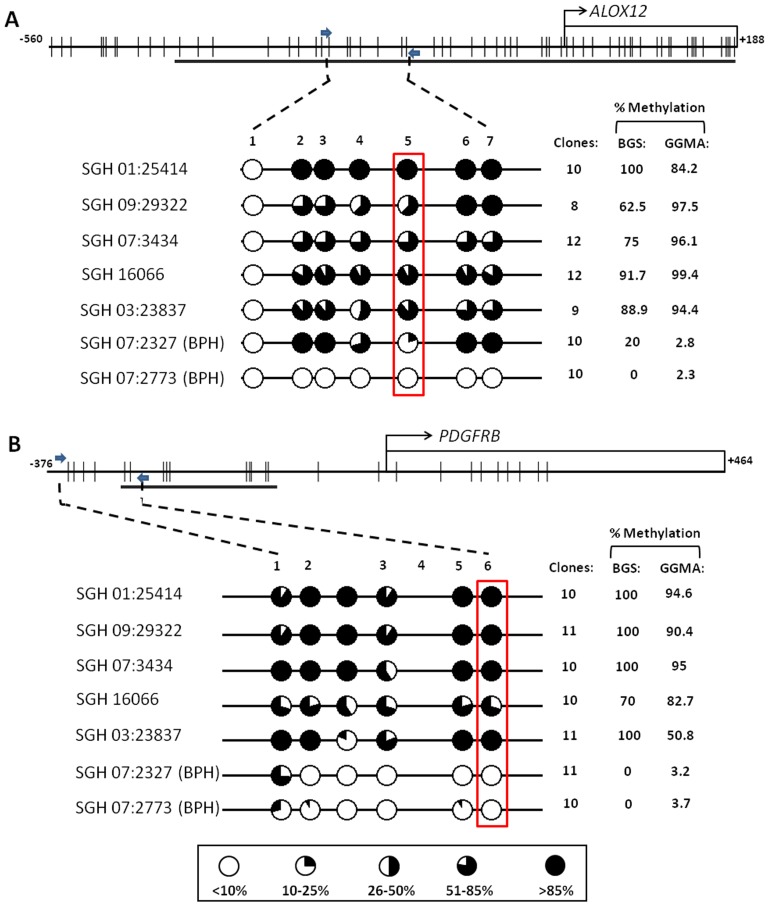
BGS of 2 genes in PHYMA. (A) *ALOX12,* and (B) *PDGFRB*. 5 prostate tumors and 2 BPH tissues were validated. The top bar shows genomics location of the gene and targeted region for sequencing (arrows). Each tick on the bar indicates the CpG site. The bottom shows the level of methylation at each site. The BGS showed good concordance with β values from the Goldengate (GGMA) assay; *ALOX12* (Spearman coefficient: 0.67), *PDGFRB* (Spearman coefficient: 0.78).

Of the 46 genes in PHYMA, we selected *ALOX12* and *PDGFRB*; 2 genes that were implicated but still relatively novel in prostate cancer, for functional validation. The objective was to ascertain β values at the coordinate array CpG in the Goldengate methylation assay (GGMA) is representative of the methylated state. BGS were carried out in *ALOX12* and *PDGFRB* with 5 prostate tumors and 2 BPH samples (Figure 3). There is good concordance between the percent methylation in samples using GGMA and BGS, comparable to those reported [25]. All prostate tumors were found to be hypermethylated in both *ALOX12* and *PDGFRB*, while the 2 BPH samples remained unmethylated (Spearman coefficient 0.67 and 0.78 respectively) (Figure 3).

## Discussion

The Gleason grading system was first advocated in 1966 because the combination of the primary and secondary grades of prostate cancer yielded better correlation with survival outcomes than a singular histological grading. More recently, reporting of tertiary Gleason grade was advised as cases with low Gleason primary and secondary grades with smaller third foci of higher Gleason grade cancers showed poorer outcomes than those with pure low Gleason score cancers [8], [42]. Inter-observer differences and experience in prostate cancer histological assessment are also factors influencing the final Gleason grading. These issues of subjectivity and inaccuracies may be minimized with better molecular genetics techniques to assess the aggressiveness of the cancer.

In this study, we identified a novel PHYMA signature that has the ability to distinguish prostate cancer from BPH and discriminate high grade cancers from low grade ones. Unlike the Gleason grading system, PHYMA is not subject to inter-observer differences in interpreting the methylation results and the archival tissues upon which the test is performed is easily obtained after surgery or biopsy without worries about material degradation or preparatory conditions. The hypermethylated signature had an accuracy of 96.4% (testing dataset) and 88.7% (OHSU dataset) in Asian and western cohorts respectively. Validations on 2 datasets showed robustness of the signature and classification model, as well as biological relevance of the genes in PHYMA in prostate cancer. In our dataset, the novel PHYMA score showed not only significant association with clinical parameters such as Gleason primary, scores, and grade, but also significant overall survival prediction after controlling for stage and Gleason indices. In the OHSU dataset, although lower PHYMA score was observed, majority of samples were classified correctly (55/62). Similar trends were observed in terms of higher PHYMA score with increasing Gleason grades and earlier biochemical recurrence although significance was not observed, which could be due to smaller sample size. Collectively, our results showed that PHYMA could potentially distinguish lethal from non-lethal prostate cancer and can be a valuable molecular tool in clinical decision of prostate cancer management.

The study is particularly relevant in contemporary prostate cancer management as it can potentially be developed into a point of care test using tissues from biopsies. This is important as more minimally invasive ablative procedures are being developed that allow patients to avoid radical prostatectomy and its attendant risks of impotence and urinary incontinence. Active surveillance with selective delayed intervention is also used in some patients with very low risk prostate cancers. However, these treatments are usually advocated for selected low risk unifocal prostate cancers and their choice are heavily reliant on the biopsy Gleason score. However, undergrading of these biopsy samples occurs in 20–30% of cases and may be minimized with better molecular interrogation techniques such as a methylation signature like PHYMA [43].

Although gene expression studies have yielded several genes and gene signatures to predict survival outcomes in prostate cancer [16], [17], these studies require the use of fresh frozen tissues under strict laboratory conditions that are difficult to replicate in busy operating room settings. The instability of RNA can be a shortcoming in the standardization of detecting changes in the RNA expression levels in microarray expression studies [44]. In contrast, the stability of DNA has proven useful in the study of methylation in prostate cancer for the predictive and prognostic clinical management of prostate cancer [45].

There are advantages in employing methylation biomarkers. A recent study has reported that there is heterogeneity in DNA methylation at both inter and intra individual level. Heterogeneity in DNA methylation with in individual was lesser than between individual and it didn’t show any correlation with gene expression. Based on these observations the authors speculate that maybe lethal metastatic cancer passes through an individual-specific clonal gate and that this clonal gate, which is observed at epigenetic level, may provide a window of opportunity to treat these cancer cell clones systematically [46]. The study explains the importance and need for more studies and markers on DNA methylation in prostate cancer.

Several methylation studies utilized a limited number of methylated genes that tend to have limited discriminatory power. Using a genome-wide approach, molecular signature elucidated to differentiate between tumors and controls can identify underlying biological factors that are more representative of the tumorigenesis or pathogenesis of the disease. For example, a panel of *GSTP1, APC, RASSF1A*, and *RARB2* has a sensitivity of 87% and specificity of 89% [47]. Another panel consisting of *GSTP1, APC,* and *RARB* has 53% sensitivity and 76% specificity [48]. Our results show that the PHYMA signature has an improved 95% sensitivity and 100% specificity for testing dataset, and 89.8% sensitivity and 66.67% specificity on the independent OHSU dataset. These results support underlying biological relevance of PHYMA signature in prostate cancer. Indeed, pathway analyses of these genes showed important and relevant pathways involved in prostate tumorigenesis, which would be difficult to elucidate with limited or small number of genes.

Validation of relatively novel genes *ALOX12* and *PDGFRB* supported the methylation microarray assay. In recent years, methylation of the LOX family of genes has been implicated in the pathogenesis of several types of cancers [49], [50], [51], [52]. In colorectal cancers, methylation of LOX have been correlated with higher microsatellite instability and *BRAF*-mutation [51], while in gastric cancer, it has been associated with tumor stage [53]. 12-Lipoxygenase or *ALOX12* is an arachidonic acid metabolizing enzyme located in the short arm of chromosome 17 that utilized the lipoxygenase (LOX) pathway. Interestingly, in acute myeloid leukaemia the hypermethylation of *ALOX12* has been associated with poorer prognosis and overall survival [49], while the demethylation of *ALOX12* observed during chemotherapy was found to be associated with treatment response [54]. *ALOX12* has also been implicated in promoting tumor progression and metastasis in prostate cancer [55], suggesting its involvement in cell proliferation and dysregulation during carcinogenesis [56]. Furthermore, in concordance to our current findings, studies carried out by Ashour et al. had demonstrated that prostate tumors with hypermethylated *ALOX12* were frequently found to be associated with poorer prognosis and increased tumor stage, supporting the role of *ALOX12* in cancer progression and metastasis.

Platelet-derived growth factor receptor-beta (*PDGFRB*) belongs to the PDGF family of growth factors that regulates cell migration, proliferation and angiogenesis [57]. In recent years, both PDGF as well as *PDGFRB* have been implicated in cancer progression, including prostate cancer [58]. Furthermore, several in vivo as well as clinical studies have also indicated that *PDGFRB* as a potential therapeutic target for metastatic disease [59]. *PDGFRB* has been shown to interact extensively with important cell signaling pathways, including *VEGF*, Ras-*MAPK*, *PI3K*, and *PLC-γ*, suggesting its important functional role in cellular regulation with the kinase family of genes [58]. *PDGFRB* expression is tightly regulated by methylation, as demonstrated by its extensive demethylated promoter region following cellular differentiation [60]. However, the role of methylation of *PDGFRB* in prostate cancer remains unclear, though it has been suggested that the PDGF methylation could regulate the activity of other oncogenic responses and act as a inducer for cellular proliferation in carcinogenesis [61].

Other important tyrosine-protein kinase family in PHYMA includes the proto-oncogene *KIT* that has been shown to be involved in cancer progression, via promoting cellular survival and proliferation [62]. Di Lorenzo et al. found a positive trend of *KIT* expression to clinical relapse in patients with prostate cancer undergoing radical prostatectomy and adjuvant hormonal therapy [63]. Although there have been few reports of *KIT* being regulated epigenetically, *in vitro* studies have indicated that cancer cell lines that tends to overexpress the *KIT* gene are often more aggressive, while cell lines that lacked *KIT* expression were hypermethylated for this gene [64]. Furthermore, the expression of *KIT* has also other epigenetic roles in controlling the methylation of other important genes like the MAGE family. Yang et al. demonstrated that the expression of *KI*T could directly influence the de-methylated status of *MAGE-A3* and *MAGE-C2*, leading to the *MAGE* gene expression [65].

There are limitations in our study. The tissue subjected to methylation studies needs pathological verification. Further cost comparison between DNA methylation and grading on routinely submitted histological specimens needs to be performed. Heterogeneity of prostate cancer may also lead to sampling issues for methylation studies. For instance, if a low grade area is used for methylation, it may erroneously lead to a favorable PHYMA score. The PHYMA signature was identified from our cohort of varying clinical stage with limited sample size. While it has been validated on a cohort of early prostate cancer, this cohort also has limited sample size and only had biochemical recurrence data. The study should be further validated in more independent cohorts, particularly in an early prostate cancer cohort with cancer-specific survival data. We are currently actively collecting more samples to further assess the 46 genes as a clinical tool and the follow up study will be reported in future. We also recognize the number of probes in our high throughput assay is also limited compared to the newer chips available now. Nevertheless, the study is an important initial evaluation of DNA methylation in an Asian cohort of prostate cancer. In elucidating PHYMA, the clinical relevance was not an initial consideration in the study design, so its significance as an independent prognostic indicator in our Asian cohort is an important outcome. It strongly supports functional relevance of the PHYMA genes in influencing overall survival.

With rapid ageing globally, the incidence of prostate cancer is expected to rise. We believe that sensitive molecular surrogates of Gleason grading will become pivotal in differentiating patients with similar Gleason grades with varied outcomes in prostate cancer, now the third most common cancer in Singapore men. Differentiating the aggressive phenotype from the latent cancers is an important step to improve patient survival while minimizing overtreatment. We believe that establishing methylated gene panels that distinguish between different prostate cancer phenotypes can help make this important distinction.

## Conclusions

Our study defined a unique prostate cancer DNA hypermethylation signature (PHYMA) that is able to distinguish prostate cancer from BPH with overall accuracies of 96.4% and 88.7% in Asian and western cohorts respectively. PHYMA scores also have the potential to differentiate cases of different Gleason primary grade and score. More importantly, it provided significant overall survival predication after controlling for Gleason indices with the potential to distinguish lethal from non-lethal prostate cancer. Further work is required to validate its application as an epigenetic surrogate for Gleason score.

## Supporting Information

File S1 **Contains tables S1, S2 & S3, and figure S1.**
(DOCX)
