# Supplementary material for: Diagnostic and Prognostic Utility of a DNA Hypermethylated Gene Signature in Prostate Cancer
Source: PLoS One. 2014 Mar 13;9(3):e91666. doi: 10.1371/journal.pone.0091666 (PMC3953552; doi:10.1371/journal.pone.0091666)

Table S1 Baseline characteristics of the western OHSU cohort.

|  | Prostate Cancer  (n=59) |
| --- | --- |
| EFS, months |  |
| Median (range) | 60(12-156) |
| Gleason Score |  |
| G6, n (%) | 23 (38.9) |
| G7, n (%) | 22 (37.3) |
| G8-G10, n (%) | 13 (22.0) |
| Stage |  |
| 1, n (%) | 28 (47.5) |
| 2, n (%) | 30 (50.8) |
| 3, n (%) | 1 (1.7) |

**Table S2 Probes and weight in the SVM model.**

| Probes | SVM Weight |
| --- | --- |
| GSTP1_E322_R | 4.47 |
| GSTM2_E153_F | 3.4 |
| GSTM2_P453_R | 3.37 |
| TRIP6_E33_F | 3.15 |
| POMC_P400_R | 3.07 |
| ALOX12_E85_R | 3.02 |
| EFNB3_E17_R | 2.98 |
| KRT5_P308_F | 2.83 |
| APC_E117_R | 2.76 |
| APC_P14_F | 2.73 |
| PEG3_E496_F | 2.59 |
| NGFR_P355_F | 2.59 |
| RAP1A_P285_R | 2.55 |
| TJP2_P330_R | 2.53 |
| TJP2_P518_F | 2.52 |
| RARB_E114_F | 2.47 |
| GSTP1_P74_F | 2.43 |
| RARB_P60_F | 2.41 |
| HTR1B_P222_F | 2.39 |
| DES_P1006_R | 2.35 |
| RARA_P1076_R | 2.34 |
| SCGB3A1_E55_R | 2.33 |
| SEPT9_P58_R | 2.29 |
| KIT_P367_R | 2.28 |
| EPHA2_P340_R | 2.28 |
| MMP14_P13_F | 2.28 |
| ACVR1_P983_F | 2.27 |
| PDGFRB_P273_F | 2.26 |
| ADAMTS12_E52_R | 2.2 |
| SCGB3A1_P103_R | 2.15 |
| RARRES1_P426_R | 2.15 |
| ZNF215_P71_R | 2.13 |
| CSF3_P309_R | 2.12 |
| SLC14A1_P369_R | 2.11 |
| IL1RN_E42_F | 2.09 |
| ELK3_P514_F | 2.06 |
| GSTM2_P109_R | 2.05 |
| CDKN1B_P1161_F | 2.03 |
| HFE_E273_R | 2.03 |
| TRIM29_P261_F | 2.01 |
| SERPINE1_E189_R | 1.98 |
| STAT5A_E42_F | 1.98 |
| MMP7_E59_F | 1.97 |
| MMP9_P189_F | 1.96 |
| ALOX12_P223_R | 1.94 |
| TIMP1_E254_R | 1.94 |
| CDH17_E31_F | 1.92 |
| MFAP4_P10_R | 1.91 |
| NEU1_P745_F | 1.91 |
| NGFB_P13_F | 1.88 |
| PYCARD_E87_F | 1.86 |
| RARA_P176_R | 1.86 |
| HOXA5_E187_F | 1.85 |
| MDR1_seq_42_S300_R | 1.8 |
| CFTR_P372_R | -1.89 |

**Table S3 Comparison of PHYMA genes with other studies.**

| Gene | Probe | Papers |
| --- | --- | --- |
| ACVR1 | ACVR1_P983_F | 2 |
| ADAMTS12 | ADAMTS12_E52_R | 2, 3 |
| ALOX12 | ALOX12_E85_R | 2 |
| ALOX12 | ALOX12_P223_R | 2 |
| APC | APC_E117_R | 2, 5, 8, 15, 16, 17, 18 |
| APC | APC_P14_F | 2, 5, 8, 15, 16, 17, 18 |
| CDH17 | CDH17_E31_F |  |
| CDKN1B | CDKN1B_P1161_F | 19 |
| CFTR | CFTR_P372_R | 2 |
| CSF3 | CSF3_P309_R | 2 |
| DES | DES_P1006_R | 2 |
| EFNB3 | EFNB3_E17_R | 2 |
| ELK3 | ELK3_P514_F | 2 |
| EPHA2 | EPHA2_P340_R | 2 |
| GSTM2 | GSTM2_E153_F | 2, 3 |
| GSTM2 | GSTM2_P453_R | 2, 3 |
| GSTM2 | GSTM2_P109_R | 2, 3 |
| GSTP1 | GSTP1_E322_R | 2,5,10,11, 15, 16, 17, 18 |
| GSTP1 | GSTP1_P74_F | 2,5,10,11, 15, 16, 17, 18 |
| HFE | HFE_E273_R | 2 |
| HOXA5 | HOXA5_E187_F | 2 |
| HTR1B | HTR1B_P222_F | 2 |
| IL1RN | IL1RN_E42_F | 2 |
| KIT | KIT_P367_R | 2 |
| KRT5 | KRT5_P308_F | 2 |
| MDR1 | MDR1_seq_42_S300_R | 8, 15, 18 |
| MFAP4 | MFAP4_P10_R | 2 |
| MMP14 | MMP14_P13_F | 2 |
| MMP7 | MMP7_E59_F | 2 |
| MMP9 | MMP9_P189_F | 2 |
| NEU1 | NEU1_P745_F | 2 |
| NGFB | NGFB_P13_F |  |
| NGFR | NGFR_P355_F | 2 |
| PDGFRB | PDGFRB_P273_F | 2 |
| PEG3 | PEG3_E496_F |  |
| POMC | POMC_P400_R | 2 |
| PYCARD | PYCARD_E87_F | 2 |
| RAP1A | RAP1A_P285_R | 2 |
| RARA | RARA_P1076_R | 2 |
| RARA | RARA_P176_R | 2 |
| RARB | RARB_E114_F | 2,5,11, 15, 16, 17, 18 |
| RARB | RARB_P60_F | 2,5,11, 15, 16, 17, 18 |
| RARRES1 | RARRES1_P426_R | 2 |
| SCGB3A1 | SCGB3A1_E55_R | 2 |
| SCGB3A1 | SCGB3A1_P103_R | 2 |
| SEPT9 | SEPT9_P58_R | 2, 3 |
| SERPINE1 | SERPINE1_E189_R | 2 |
| SLC14A1 | SLC14A1_P369_R | 2 |
| STAT5A | STAT5A_E42_F | 2 |
| TIMP1 | TIMP1_E254_R | 2 |
| TJP2 | TJP2_P330_R | 2, 3 |
| TJP2 | TJP2_P518_F | 2, 3 |
| TRIM29 | TRIM29_P261_F | 2 |
| TRIP6 | TRIP6_E33_F | 2 |
| ZNF215 | ZNF215_P71_R | 2 |

Legend:

| **Paper** | **Title** | **Author** | **Publication** | **Year** |
| --- | --- | --- | --- | --- |
| 1 | Gene Expression Alterations in Prostate Cancer Predicting Tumor Aggression and Preceding Development of Malignancy | Yu et al | Journal of Clinical Oncology | 2004 |
| 2 | DNA methylation profiling reveals novel biomarkers and important roles for DNA methyltransferases in prostate cancer | Kobayashi et al | Genome Research | 2011 |
| 3 | A DNA methylation microarray-based study identifies ERG as a gene commonly methylated in prostate cancer | Schwartzman et al | Epigenetics | 2011 |
| 4 | EFEMP1 as a Novel DNA Methylation Marker for Prostate Cancer: Array-based DNA Methylation and Expression Profiling | June et al | American Association for Cancer Research | 2011 |
| 5 | Multicenter Evalutation of an Investigational Prostate Cancer Methylation Assay | Baden et al | The Journal of Urology | 2009 |
| 6 | Molecular Classification of prostate cancer using curated expression signatures | Markert et al | PNAS | 2011 |
| 7 | Gene Expression correlates of clinical prostate cancer behavior | Singh et al | Cancer Cell | 2002 |
| 8 | Methylated genes as potential biomarkers in prostate cancer | Veronique et al | BJU International | 2010 |
| 9 | Discovery of Novel Hypermethylated Genes in Prostate Cancer Using Genomic CpG Island Microarray | Kron et al | PLoS ONE | 2008 |
| 10 | High throughput screening of methylation status of genes in prostate cancer using an oligonucleotide methylation array | Yu et al | Carcinogenesis | 2005 |
| 11 | Global methylation profiling for risk prediction of prostate cancer (T vs N) | Mahapatra et al | Clinical Cancer Research | 2012 |
| 12 | Global methylation profiling for risk prediction of prostate cancer (Recurrence vs nonrecurrence) | Mahapatra et al | Clinical Cancer Research | 2012 |
| 13 | Global methylation profiling for risk prediction of prostate cancer (clinical recurrence vs biochemical recurrence) | Mahapatra et al | Clinical Cancer Research | 2012 |
| 14 | Global methylation profiling for risk prediction of prostate cancer (systemic recurrence vs local recurrence) | Mahapatra et al | Clinical Cancer Research | 2012 |
| 15 | Epigenetic biomarkers in urological tumors: A systematic review | Jerónimo et al | Cancer Letters | 2011 |
| 16 | The Epigenetic Promise for Prostate Cancer Diagnosis | Neste et al | The Prostate | 2012 |
| 17 | Epigenetic biomarkers in prostate cancer: Current and future uses | Chiam et al | Cancer Letters | 2012 |
| 18 | Biomarkers in prostate cancer epidemiology | Verma et al | Cancers | 2011 |
| 19 | Loss of CDKN1B/p27Kip1 expression is associated with ERG fusion-negative prostate cancer, but is unrelated to patient prognosis. | Sirma et al | Oncology Letters | 2013 |

**Figure S1 Clinical relevance in OHSU western cohort. (a) Tumor samples of Gleason score 4-8 harbor higher PHYMA score. (b) Univariate biochemical recurrence shows trend that higher PHYMA samples tend to have earlier recurrence but no significance was observed.**


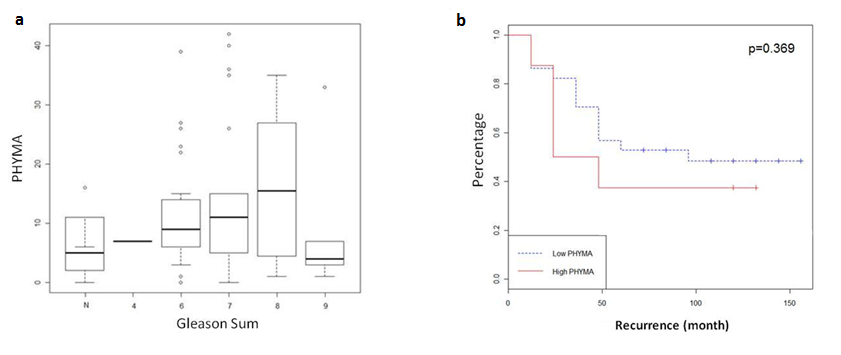

Supplement: File S1 — Contains tables S1, S2 & S3, and figure S1. (DOCX) [file pone.0091666.s001.docx]
